# Polycystic ovary syndrome and psychological distress: Urban–rural comparison among reproductive-aged women in Bangladesh

**DOI:** 10.1371/journal.pgph.0004937

**Published:** 2025-12-31

**Authors:** Anup Talukder, Tahmina Akter Tithi, Maruf Hasan Rumi, Abdul Muyeed

**Affiliations:** 1 Department of Statistics, Jatiya Kabi Kazi Nazrul Islam University, Mymensingh, Bangladesh; 2 Department of Public Administration, University of Dhaka, Dhaka, Bangladesh; University of Tunis El Manar Faculty of Medicine of Tunis: Universite de Tunis El Manar Faculte de Medecine de Tunis, TUNISIA

## Abstract

Polycystic ovary syndrome (PCOS) is a common hormonal disorder of reproductive-aged women all over the world. Low- and middle-income countries, such as Bangladesh, often give inadequate attention to mental health issues. As a result, a growing number of women with PCOS are dealing with mental health issues, including an under diagnosis and under-treatment of psychological illnesses. The study assesses the prevalence and psychological impacts of PCOS among women in the reproductive age as well as compared the mental health inequalities between rural and urban women in Bangladesh. A sample of 212 women in the reproductive age was collected using a convenience sampling procedure. Additionally, for diagnosis of PCOS, the Rotterdam criteria were used. Depression, anxiety, stress, and insomnia were measured using DASS-21 and ISI-7 tool. To analyze the association and impact of PCOS on mental health problems, a chi-square test and multivariate binary logistic regression were conducted. The Mann-Whitney U test was implemented to assess mental health disparities among rural-urban reproductive women. Among the participants, the PCOS prevalence was 50.9%, and the rates of depression, anxiety, stress, and insomnia were 51.4%, 62.3%, 46.7%, and 53.3%, respectively. Based on the Rotterdam criteria, women with PCOS had a significantly greater prevalence of psychological disorders than women without PCOS. Women residing in rural region were more prone to meet Rotterdam criteria and had higher anxiety and insomnia syndrome. Women who met two or three diagnostic criteria had a higher chance of experiencing psychological distress than others. Furthermore, relationship status and BMI are useful predictive factors of mental health consequences. The findings showed a substantial prevalence of emotional distress among PCOS women in Bangladesh. Additionally, a comprehensive mental health investigation is suggested for PCOS. Addressing both the physical and psychological aspects of PCOS is essential to develop women’s overall well-being and quality of life.

## 1. Introduction

Polycystic ovary syndrome (PCOS) is one of the most prevalent endocrine disorders among reproductive-aged women worldwide [1]. This disorder produces extreme amount of androgen from the ovaries because of its multidimensional and polygenic behavior. It also comprises dysregulation of CYP11a gene and upregulation of the enzymes involved in androgen biosynthesis [2]. Furthermore, it is also linked to the insulin receptor gene, located on the chromosome 19p13. 2 [3]. According to the World Health Organization, it affects between 6 and 10% of reproductive-age women and an estimated 70% of cases are undiagnosed worldwide. This syndrome has large influence on the female biological aspect such as obesity, body image and infertility, leading to social stigma. PCOS has an adverse affect on psychological health, contributing to depression, anxiety, stress, and insomnia in women worldwide who are of reproductive age [4]. The worldwide prevalence of PCOS is estimated to be 9.2% [5]. A prevalence of 11.4% has been reported in the south Asian region [6]. However, the incidence of PCOS and subsequent mental health disorders in South Asian women appears to be substantially higher than the worldwide average. This is due to some reasons such as social and cultural taboo, religious and political diversity, collectivist society, and a shortage of mental health services [7–9]. Moreover, PCOS women were more likely to contribute certain physical syndromes, include menstrual irregularity or infertility, obesity, insulin resistance, dyslipidaemia, hypertension and metabolic dysfunction rather than PCOS free, which may subsequently contribute to cardio metabolic disorders [10]. Despite the physical issues, it has also significant association with some psychological illnesses such as depression, anxiety, stress, insomnia, bipolar disorder, obsessive compulsive disorder, and somatization disorders [11,12]. Other frequently reported symptoms include concentration difficulty, low self-esteem, loss of interest or pleasure, fatigability, increased fatigue, reduced activity, appetite decrease and possible suicidal ideation [13]. The relationship between mental health condition and PCOS implies that increase in prevalence of PCOS may worsen the circumstance of mental health.

In South and Southeast Asian nations, including India and Thailand, studies have shown that women with PCOS endure comparable psychological distress, often exacerbated by insufficient healthcare access and societal stigmas. Keeratibharat et al. (2024) conducted a study revealing that the prevalence of depression, anxiety, and poor mental well-being among Thai women with PCOS was 3.8%, 11.9%, and 16.9%, respectively [14]. Studies in India have shown that women with PCOS have high levels of anxiety and depression because they can’t get the treatment they need [9].

Nonetheless, owing to a lack of resources and information, developing nations like Bangladesh are confronting the challenge of mental health burdens [15]. Hasan et al. (2024) executed a cross-sectional study involving 409 PCOS patients, revealing prevalence rates of loneliness, anxiety, and depressive syndrome at 71%, 88%, and 60%, respectively [16]. Additionally, Ishrat et al. (2021) recruited 126 consecutive infertile women with PCOS for a cross-sectional study, demonstrating that obesity correlates with hyperandrogenism, hyperinsulinemia, insulin resistance, and metabolic syndrome [17]. Furthermore, a cross-sectional study by Kamrul-Hasan et al. (2021) on adolescents with PCOS revealed that an elevated BMI heightens the risk of metabolic syndrome [18]. Sarker et al. (2015) reported in their study that the greatest clinical predictor for PCOS diagnosis in infertile women is the presence of hirsutism [19]. Numerous cultural and societal variables exacerbate the problems associated with PCOS in low- and middle-income nations like Bangladesh [20,21]. Consequently, a large number of women experience numerous chronic mental health problems as a result of being undetected and untreated. Despite the fact that a great deal of research has been done globally on the psychological implications of PCOS, Bangladesh has gotten less attention.

Existing research has investigated the impact of PCOS on mental health focusing on depressive syndrome, anxiety, loneliness as well as its clinical, hormonal and metabolic parameters. However, few researches have thoroughly examined the psychological problems that PCOS patients in Bangladesh face, such as stress and insomnia. The aim of this study was to assess the prevalence of polycystic ovary syndrome (PCOS) and associated psychological distress, including depression, anxiety, stress, and insomnia, among women in reproductive age in Bangladesh, as well as to evaluate the effect of PCOS on these outcomes. The specific objectives of the study were to compare the prevalence of psychological distress between women with and without PCOS, and to examine differences in psychological outcomes between urban and rural women. By addressing these objectives, the study seeks to clarify the psychological burden faced by women with PCOS and reinforce the necessity for comprehensive treatment by examining the prevalence and possible causes of these issues.

## 2. Materials and methods

### 2.1. Ethics statement

Ethical approval for this study was granted by the Research Ethics Committee of the Public Health Foundation Bangladesh (PHFBD) in Dhaka. The study adhered to the ethical principles outlined in the Declaration of Helsinki. Written informed consent was obtained from all participants prior to data collection. The research protocol was approved under reference number PHFBD-ERC-NFP-E-18/2025.

At the beginning of data collection, participants received a thorough explanation of the study’s objectives, aims, methods, affiliations, benefits, and hazards at the planned face to face interview session. Afterward, the respondents agreed to take part in the study, and the data collectors promised that their answers would be kept private and not shared. With their consent, data was subsequently gathered from them for the research purpose.

### 2.2. Study design

A cross-sectional comparative study design was used to investigate the relationship between psychological distress and PCOS. Due to its ability to quantify exposure like PCOS diagnosis and psychological outcomes simultaneously within a defined population, this design was selected to enable group comparisons without requiring long-term follow-up. Similar designs have been effectively used in related studies examining PCOS and mental health. Hasan et al. (2024) and Kite et al. (2021) utilized comparable methodologies in their investigations on associated subjects [16,22].

### 2.3. Study participants and sample

The sample was collected from the women in Bangladesh who were of reproductive age. Although reproductive age was defined as 15–49 years, all respondents who participated were aged 18 years or above; no participants younger than 18 years were included in the final sample [23], regardless of whether they had PCOS or not. The sample size was calculated using Godden’s algorithm with a 5% margin of error and a 5% threshold of significance (α = 0.05). A cross-sectional survey that found an 11.92% prevalence of anxiety among Thai women with PCOS was used to calculate the study’s sample size [14].


$$n=\frac{z^{\text{2}}pq}{\begin{array}{c} e^{\text{2}} \end{array}}=\frac{{\left(\text{1}.\text{9}\text{6}\right)}^{\text{2}}\times \text{0}.\text{1}\text{1}\text{9}\text{2}\times \text{0}.\text{8}\text{8}\text{0}\text{8}}{\begin{array}{c} {\left(\text{0}.\text{0}\text{5}\right)}^{\text{2}} \end{array}}=\text{1}\text{6}\text{1}$$

**Fig 1:**
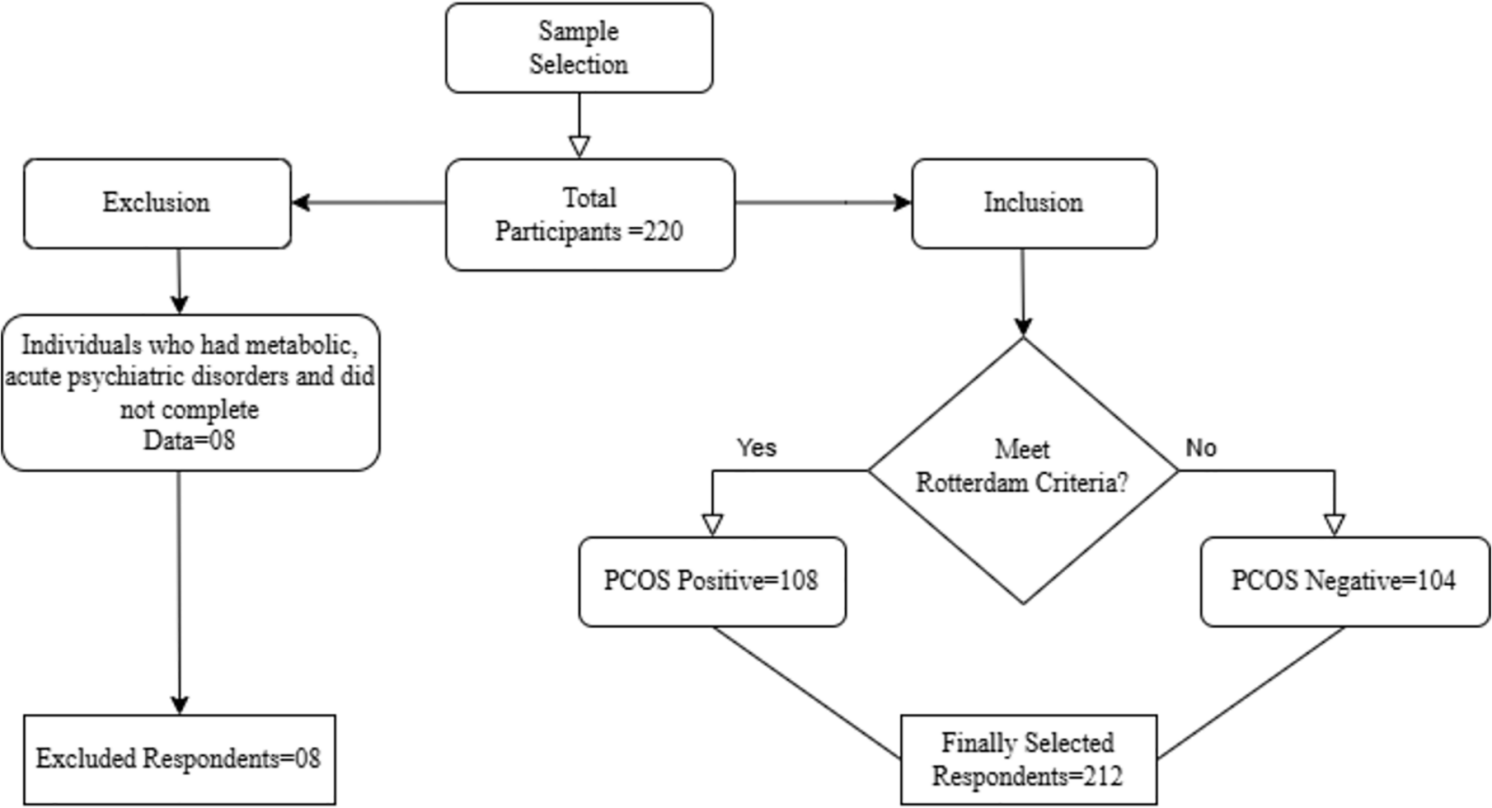
Sampling Flow chart with study inclusion and exclusion criteria.

It was determined that 161 was the ideal sample size. 220 reproductive female participants were given the questionnaire; regardless of whether they had PCOS or not. After excluding eight people with metabolic abnormalities, hormonal medication, acute psychiatric concerns, and incomplete data, 212 participants were ultimately chosen for the study with their consent. Out of the 212 respondents who had been selected, 108 were diagnosed with PCOS after meeting at least two of the Rotterdam criteria. Among 108 PCOS patients, 39 respondents only satisfied two of the Rotterdam criteria, whereas 69 respondents met all three criteria. However, since they satisfied fewer than two diagnostic criteria, the other 104 respondents were categorized as not having PCOS. In Fig 1, a sampling flowchart was displayed.

A post-hoc power analysis was conducted to evaluate whether the adequate sample size of 108 participants with PCOS and 104 without, had sufficient power to detect group differences in psychological distress. The findings indicated an estimated power of approximately >95%, suggesting that the sample size was sufficient to detect an odds ratio of 4.6. However, the power was considerably lower for detecting smaller effect sizes. Moreover, a large effect of 72% was found through calculating the magnitude of effect size using Cohen’s h, suggesting that the study was highly powered. Therefore, the optimal sample sizes provided sufficient power to evaluate moderate-to-large relationships.

### 2.4. Instrument and data collection

The participants were chosen using a convenience sampling method from the gynecology outpatient departments of four government hospitals in the Mymensingh division. The data was collected between March 1, 2025, and April 15, 2025. In Bangladesh, reproductive health conditions are almost the same in every administrative division [24]. For convenience, the study was carried out in Mymensingh with the goal of cutting down on research expenses and time. Data were collected from four government hospitals located in four administrative districts within the Mymensingh division. The institutions comprise Mymensingh Medical College and Hospital, Netrokona Medical College and Hospital, Jamalpur Sadar Hospital, and Sherpur Sadar Hospital. To ensure representation from all four districts of Mymensingh division, we decided prior to data collection to recruit approximately equal numbers of participants (50 each) from each hospitals. A slightly larger number (62) was obtained from Mymensingh Medical College and Hospital, which is the division’s largest referral centre with a higher amount of patients. This quota-based strategy was used prior to data collection, driven by the necessity for equitable representation across sites and logistical feasibility such as time, resources, staff availability. The data collectors collected the relevant data for the study conveniently from reproductive-aged women exiting the gynecology departments who met the inclusion criteria and gave consent. Data was gathered by conducting an in-person interview and recording the answers with a structured questionnaire. Participants received printed versions of the questionnaires. A validated four-section questionnaire was created in Bengali and English. To make sure the survey was valid and reliable; a pilot study with ten respondents was carried out. The Rotterdam criteria, which are the diagnostic criteria for PCOS, are incorporated into the questionnaire in the first section. Hyperandrogenism, irregular menstruation, and polycystic ovaries on ultrasound are the three Rotterdam criteria for diagnosing PCOS. At least two of these three criteria had to be met in order to diagnose PCOS. Background information was provided in the second section. For ensuring consistency, PCOS diagnosis was confirmed by qualified gynecologists at each of the four participating hospitals. Clinicians confirm that clinical factors were used to assess hyperandrogenism, particularly hirsutism, acne, and androgenic alopecia which was reported by participants. Due to limited resources, biochemical testing for androgen levels was not conducted. Under the Rotterdam criteria, irregular menstruation was classified as either oligomenorrhea (cycles longer than 35 days), amenorrhea (absence of menstruation for more than 90 days), or persistent irregular periods (less than 8 cycles annually). Ultrasound was used to confirm polycystic ovaries when each ovary had ≥ 12 follicles measuring 2–9 mm or when the ovarian volume was greater than 10 cm³. The Depression, Anxiety, and Stress Scale (DASS-21) was used in the third segment, and the Insomnia Severity Index (ISI-7) was used in the last.

### 2.5. Depression, anxiety, stress scale-21 (DASS-21)

Depression, Anxiety, and Stress Scale (DASS-21) is used to assess participants’ overall psychological well-being over the past week. The 21 questions on the DASS-21 are composed of seven items from the subscales for stress, anxiety, and depression. A 4-point Likert scale with a range of 0–3 is used with a sequence of statements to show the degree of agreement. The following is the definition of the rating scale: The numbers 0–3 represent not at all, some of the time, good part of the time, and most of the time, respectively. The relevant category scores are summed together, and the overall score is then multiplied by two to approximate the degrees of stress, anxiety, and depression. The ranges of depression are determined as (0–9), (10–13), (14–20), (21–27), and 28 + which demonstrates the intensity of normal, mild, moderate, severe, and extremely severe, respectively. A normal level of anxiety is indicated by a total score between 0 and 7. Furthermore, mild, moderate, severe, and extremely severe anxiety are represented by the score ranges of 8–9, 10–14, 15–19, and 20 + , respectively. Four different levels are identified in the total score range for stress: (0–14), (15–18), (19–25), (26–33), and 34 + . These levels represent normal, mild, moderate, severe, and extremely severe stress, respectively [25]. A detrimental state for stress, anxiety, and depression is indicated by scores that range from moderate to extremely severe. In order to ensure the equivalence of the translation, a faculty member from English department of Jatiya Kabi Kazi Nazrul Islam University verified the Bengali translation of the original DASS-21 scale as part of the language translation procedure. In the pilot study, the Cronbach’s α coefficient of the scale was 0.920, which indicated high internal consistency and reliability. The final version of the tool retained this level of reliability after data collection.

### 2.6. Insomnia severity index (ISI-7)

A sleep disorder called insomnia makes it difficult to fall asleep. Additionally, chronic insomnia has been linked to a number of major health concerns, such as metabolic syndrome, type 2 diabetes mellitus, cardiovascular disease, weight-related difficulties, hypertension, dyslipidemia, and colorectal cancer [26]. The insomnia severity index is a widely used measurement tool to assess the degree of sleep-related problems over the previous two weeks. Seven items with a five-point Likert scale comprise the evaluation. The insomnia total score ranges from 0 to 28. Insomnia is more severe when the score is more than 14. Chronic insomnia is indicated by a higher total insomnia score. The sum of the scores from all seven questions is used to determine how severe insomnia is. The following is an explanation of how the overall scores were categorized: Clinically severe insomnia is absent when the overall score falls between 0 and 7. The overall scores fall into three categories: “sub-threshold”, “moderate”, and “severe clinical insomnia” [27]. These are 8–14, 15–21, and 22–28, respectively. The scale’s Cronbach’s α coefficient in the pilot test was 0.923, indicating strong reliability and internal consistency. After data collecting, the tool’s final version retained this degree of reliability. A professor from Jatiya Kabi Kazi Nazrul Islam University’s English department verified the Bengali translation of the original ISI scale as part of the validation procedure.

### 2.7. Statistical analysis

The research employed descriptive statistics to illustrate categorical variables, emphasizing frequency and percentage. The Depression, Anxiety, Stress Scale-21 (DASS-21) and the Insomnia Severity Index-7 (ISI-7) were utilized to measure the severity of mental health issues, including depression, anxiety, and stress, as well as insomnia. Additionally, Cronbach’s *α* coefficient of internal consistency was utilized to assess the reliability of DASS-21 and ISI-7 [28]. The ideal cut-off for Cronbach’s *α* is 0.70, with the suggested range extending from zero to one. A value approaching 1 and exceeding 0.70 signifies improved internal consistency or reliability [29]. To examine the association between PCOS, emotional distress, and socio-demographic characteristics, a bivariate chi-square analysis was performed. The influence of PCOS on mental health problems and insomnia was then to be clarified by examining the cause-and-effect relationship of significant related factors using multivariate binary logistic regressions (MBLR) with a 95% confidence interval. Furthermore, the Mann-Whitney U test was used to evaluate the disparities in depression, anxiety, stress, and insomnia between Bangladeshi women residing in urban and rural areas. Before conducting the Mann-Whitney test, the assumption of distribution symmetry between groups was checked using QQ plots. As a result, the finding ensured that the distribution was similarly shaped that validated the use of Mann Whitney test for comparing median. All forms of statistical analysis were carried out using IBM Incorporation’s Statistical Package for Social Science (SPSS, version 25). Additionally, R programming software (version 4.5.1) was used to create the prevalence graph and post-hoc power analysis was carried out using G*Power tools (version 3.1.9.7) to check the ability whether the adequate sample size had sufficient power to detect group differences.

## 3. Results

Table 1 depicts social demographic and health related characteristics among reproductive aged women in Bangladesh. The study involved a total participant with an average age of 24.04 (*±*3.074) years, an average height of 166.03 (*±*14.586) inches, and a mean weight of 63.405 (*±*20.120) kg. Most respondents are students (81.6%) with honors-level education (63.2%). A majority of them are single (56.6%) and live in urban areas (53.3%). On the other hand, BMI is equally divided between underweight and normal weight categories (33% each). On average, participants reported 14.73 (*±*5.869) levels of depression, 15.78 (*±*7.410) levels of anxiety, 18.14 (*±*8.411) levels of stress, and 14.43 (*±*5.317) levels of insomnia. Most of the participants have PCOS present (50.9%) than absent (49.1%), while depression (51.4%), anxiety (62.3%), stress (46.7%), and insomnia (53.3%) are prevalent.

**Table 1:** Social demographic and Health related characteristics among reproductive aged women in Bangladesh.

Factor/Variable	Category/Level	Percentage n (%)
**Age**	18-25	175(82.5)
	26-35	34(16.0)
	36-45	3(1.4)
**Occupation**	Student	173(81.6)
	Employee	16(7.5)
	Housewife	23(10.8)
**Educational Qualification**	Primary & secondary	7(3.3)
	Higher Secondary	34(16.0)
	Honors’	134(63.2)
	Masters/post-graduation	37(17.5)
**Relationship Status**	Single	120(56.6)
	Engaged in relationship	48(22.6)
	Married	44(20.8)
**Residential Area**	Urban	113(53.3)
	Rural	99(46.7)
**Any Disease**	No	134(63.2)
	Yes	33(15.6)
	Do not know	45(21.2)
**Physical disability**	No	191(90.1)
	Yes	7(3.3)
	Don’t know	14(6.6)
**Friendly Family Environment**	No	19(9.0)
	Yes	146(68.9)
	Maybe	47(22.2)
**BMI**	Underweight (Below 18.5)	70(33.0)
	Normal weight (18.5-24.9)	70(33.0)
	Overweight (25.0-29.9)	46(21.7)
	Obese (30.0 an above)	26(12.3)
**Parity**	0	13(6.1)
	1	199(93.9)
**Age of menarche**	<12	30(14.2)
	>=12	182(85.8)
**Smoking and drinking status**	No	179(84.4)
	Yes	17(8.0)
	Sometimes	16(7.5)
**PCOS diagnosis**	Present	108(50.9)
	Absent	104(49.1)
**Depression**	Yes	109(51.4)
	No	103(48.6)
**Anxiety**	Yes	132(62.3)
	No	80(37.7)
**Stress**	Yes	99(46.7)
	No	113(53.3)
**Insomnia**	Yes	113(53.3)
	No	99(46.7)
**Age**	Mean (*±SD*)	24.04 (3.074)
**Height (in inch)**	Mean (*±SD*)	166.03 (14.586)
**Weight (in Kg)**	Mean (*±SD*)	63.405 (20.120)
**Age of menarche**	Mean (*±SD*)	12.91 (1.261)
**Depression**	Mean (*±SD*)	14.73 (5.869)
**Anxiety**	Mean (*±SD*)	15.78 (7.410)
**Stress**	Mean (*±SD*)	18.14 (8.411)
**Insomnia**	Mean (*±SD*)	14.43 (5.317)

Table 2 depicts factor-wise associations between PCOS, socio-demographic variables and depression, anxiety, stress, and insomnia. Based on the residential areas of the respondents, rural women had higher levels of anxiety (p < 0.001) and stress (p < 0.05), while urban women experienced lower insomnia (p < 0.01). Furthermore, depression (p < 0.001) and anxiety (p < 0.05) were significantly associated with obesity (BMI ≥ 30). On the other hand, women with PCOS present had significantly higher levels of depression, anxiety, stress and insomnia compared to those absent (p < 0.001). Additionally, women meeting two or three Rotterdam criteria exhibited significantly higher rates of depression, anxiety, stress, and insomnia (p < 0.001).

**Table 2:** Factor-wise associations between PCOS, socio-demographic variables and depression, anxiety, stress, and insomnia.

Variables	Depression			Anxiety			Stress			Insomnia		
	Yes (%)	No (%)	p-value	Yes (%)	No (%)	p-value	Yes (%)	No (%)	p-value	Yes (%)	No (%)	p-value
**Socio-demographics**												
**Age**												
18-25	79.8	85.4	0.191	82.6	82.5	0.986	82.8	82.3	0.751	85.8	78.8	0.377
26-35	17.4	14.6		15.9	16.3		15.2	16.8		13.3	19.2	
36-45	2.8	0.0		1.5	1.3		2.0	0.9		0.9	2.0	
**Occupation**												
Student	78.0	85.4	0.283	78.8	86.3	0.241	78.8	84.1	0.593	85.0	77.8	0.061
Employee	10.1	4.9		7.6	7.5		9.1	6.2		3.5	12.1	
Housewife	11.9	9.7		13.6	6.3		12.1	9.7		11.5	10.1	
**Educational Qualification**												
Primary & secondary	3.7	2.9	0.702	3.0	3.8	0.450	3.0	3.5	0.883	2.7	4.0	0.515
Higher Secondary	14.7	17.5		18.9	11.3		18.2	14.2		16.8	15.2	
Honours	61.5	65.0		59.8	68.8		61.6	64.6		66.4	9.6	
Masters/post-graduation	20.2	14.6		18.2	16.3		17.2	17.7		14.2	21.2	
**Relationship Status**												
Single	51.4	62.1	0.05*	57.6	55.0	0.593	55.6	57.5	0.959	54.9	58.6	0.851
Engaged in relationship	29.4	15.5		20.5	26.3		23.2	22.1		23.9	21.2	
Married	19.3	22.3		22.0	18.8		21.2	20.4		21.2	20.2	
**Residential Area**												
Urban	46.8	46.6	0.978	37.1	62.5	0.000***	38.4	58.0	0.023**	38.1	56.6	0.007**
Rural	53.2	53.4		62.8	37.5		61.6	46.0		61.9	43.4	
**Any Disease**												
No	67.0	59.2	0.299	63.6	62.5	0.439	63.6	62.8	0.987	62.8	63.6	0.713
Yes	11.9	19.4		17.4	12.5		15.2	15.9		14.2	17.2	
Do not know	21.1	21.4		8.9	25.0		21.2	21.2		23.0	19.2	
**Physical disability**												
No	89.9	90.3	0.949	91.7	87.5	0.586	88.9	91.2	0.820	91.2	88.9	0.820
Yes	3.7	2.9		3.0	3.8		4.0	2.7		2.7	4.0	
Don’t know	6.4	6.8		5.3	8.8		7.1	6.2		6.2	7.1	
**Friendly Family Environment**												
No	10.1	7.8	0.804	11.4	5.0	0.286	11.1	7.1	0.407	8.0	10.1	0.635
Yes	68.8	68.9		66.7	72.5		64.6	72.6		71.7	65.7	
Maybe	21.1	23.3		22.0	22.5		24.2	20.4		20.4	24.2	
**BMI**												
Underweight (Below 18.5)	26.6	39.8	0.000***	32.6	33.8	0.045*	37.4	29.2	0.089	32.7	33.3	0.170
Normal weight (18.5-24.9)	35.8	30.1		39.4	22.2		37.4	29.2		31.9	34.3	
Overweight (25.0-29.9)	32.1	10.7		17.4	28.7		15.2	27.4		26.5	16.2	
Obese (30.0 an above)	5.5	19.4		10.6	15.0		10.1	14.2		8.8	16.2	
**Parity**												
0	4.6	7.8	0.335	6.1	6.3	0.956	5.1	7.1	0.539	6.2	6.1	0.968
1	95.4	92.2		93.9	93.8		94.9	92.9		93.8	93.9	
**Age of menarche**												
< 12	14.7	13.6	0.821	16.7	10.0	0.177	14.1	14.2	0.997	15.0	13.1	0.690
>=12	85.3	86.4		83.3	90.0		85.9	85.8		85.0	86.9	
**Smoking and drinking status**												
No	82.6	86.4	0.342	82.6	87.5	0.524	83.8	85.0	0.961	85.0	83.8	0.459
Yes	7.3	8.7		8.3	7.5		8.1	8.0		6.2	10.1	
Sometimes	10.1	4.9		9.1	5.0		8.1	7.1		8.8	6.1	
**PCOS Diagnosis**												
PCOS Absent	27.5	71.8	0.000***	31.8	77.5	0.000***	19.2	75.2	0.000***	33.6	66.7	0.000***
PCOS Present	72.5	28.2		68.2	22.5		80.8	24.8		66.4	33.3	
**Depression status**												
No	–	–	–	49.2	47.5	0.806	27.3	67.3	0.000***	41.6	56.6	0.030**
Yes	–	–		50.8	52.5		72.7	32.7		58.4	43.4	
**Anxiety status**												
No	38.5	36.9	0.806	–	–	–	32.3	42.5	0.128	29.2	47.5	0.006**
Yes	61.5	63.1		–	–		67.7	57.5		70.8	52.5	
**Stress status**												
No	33.9	73.8	0.000***	49.2	60.0	0.128	–	–	–	46.9	60.6	0.046*
Yes	66.1	26.2		50.8	40.0		–	–		53.1	39.4	
**Insomnia status**												
No	39.4	54.4	0.030*	39.4	58.8	0.006**	39.4	53.1	0.046*	–	–	–
Yes	60.6	45.6		60.6	41.3		60.6	46.9		–	–	
**Rotterdam Criteria**												
Did not meet any Rotterdam criteria	27.5	71.8	0.000***	31.8	77.5		19.2	75.2		33.6	66.7	
Meet only two Rotterdam criteria	32.1	3.9		22.7	11.3	0.000***	34.3	4.4	0.000***	21.2	15.2	0.000***
Meet three Rotterdam criteria	40.4	24.3		45.5	11.3		46.5	20.4		45.1	18.2	

Note.*p < 0.05, **p < 0.01, ***p < 0.001.

Table 3 illustrates the binary logistic regression analysis for factors associated with depression, anxiety, stress, and insomnia. According to the table, being engaged in a relationship increases the likelihood of depression (aOR=2.676) with a significant association (p < 0.05). In addition, normal weight increases the risk for depression (aOR=2.450, p < 0.05) and anxiety (aOR=2.601; p < 0.05), while overweight individuals are more likely to experience depression significantly (aOR=8.802, p < 0.001). On the other hand, such mental health issues are associated with having PCOS (all conditions, p < 0.001). However, meeting two or three Rotterdam criteria is significantly associated with all four mental health issues.

**Table 3:** Binary Logistic Regression Analysis of Factors Associated with Emotional Distress.

Variables	Depression			Anxiety			Stress			Insomnia		
	aOR^a^	95% CI	p-value	aOR^a^	95% CI	p-value	aOR^a^	95% CI	p-value	aOR^a^	95% CI	p-value
Socio-demographics												
**Relationship Status**												
Single	Ref.^b^			Ref.								
Engaged in relationship	2.676	(1.102,6.496)	0.030*	–	–	–	–	–	–	–	–	–
Married	0.835	(0.336,2.076)	0.699	–	–	–	–	–	–	–	–	–
**Residential Area**												
Urban				Ref.			Ref.			Ref.		
Rural	–	–	–	1.749	(0.922,3.321)	0.087	0.969	(0.466,2.019)	0.934	1.462	(0.804,2.657)	0.213
**BMI**												
Underweight (Below 18.5)	Ref.			Ref.								
Normal weight (18.5-24.9)	2.450	(1.061,5.655)	0.036*	2.601	(1.102,6.140)	0.029*	–	–	–	–	–	–
Overweight (25.0-29.9)	8.802	(3.097,25.016)	0.000***	0.477	(0.187,1.213)	0.120	–	–	–	–	–	–
Obese (30.0 and above)	0.323	(0.096, 1.092)	0.069	0.423	(0.140,1.280)	0.128	–	–	–	–	–	–
**PCOS Diagnosis**												
PCOS Absent	Ref.			Ref.			0.097	(0.043,0.217)	0.000***	Ref.		
PCOS Present	5.029	(2.151,11.762)	0.000***	6.318	(3.264,12.231)	0.000***	Ref.			3.519	(1.936,6.395)	0.000***
**Depression, anxiety, stress, insomnia**												
**Depression status**												
No							Ref.			Ref.		
Yes	–	–	–	–	–	–	2.744	(1.371,5.491)	0.004**	1.316	(0.650,2.664)	0.445
**Anxiety status**												
No										Ref.		
Yes	–	–	–	–	–	–	–	–	–	1.246	(0.619,2.509)	0.537
**Stress status**												
No	Ref.									Ref.		
Yes	4.288	(1.874,9.813)	0.001**	–	–	–	–	–	–	0.778	(0.362,1.670)	0.519
**Insomnia status**												
No	Ref.			Ref.			Ref.					
Yes	0.887	(0.427,1.843)	0.749	1.262	(0.611,2.608)	0.530	0.732	(0.351,1.526)	0.406	–	–	–
**Rotterdam Criteria**												
Did not meet any Rotterdam criteria	Ref.^b^			Ref.			Ref.			Ref.		
Meet only two Rotterdam criteria	21.583	(7.056,66.022)	0.000***	4.921	(2.121,11.416)	0.000***	30.421	(10.514,88.016)	0.000***	2.779	(1.301,5.934)	0.008**
Meet three Rotterdam criteria	4.341	(2.269,8.306)	0.000***	9.841	(4.410,21.961)	0.000***	8.947	(4.418,18.119)	0.000***	4.921	(2.520,9.611)	0.000***

Note. Adjusted odd ratio^a^, ^b^Reference group.*p < 0.05, **p < 0.01, ***p < 0.001.

Table 4 demonstrates the association of Rotterdam criteria across residential areas. The table shows significant differences in the association of Rotterdam criteria between urban and rural areas (p < 0.001). On the other hand, meeting only two Rotterdam criteria had 2.5 times higher odds (aOR = 2.497) of meeting the criteria compared to those who did not, with a significant (p < 0.05) in rural areas. Additionally, meeting all three Rotterdam criteria had 5.3 times higher odds (aOR = 5.313) with a highly significant association (p < 0.001).

**Table 4:** Association of Rotterdam Criteria with Residential Area: Chi-Square and Logistic Regression Analysis.

Chi-square				Binary Logistic Regression		
Variables	Residential Area		p- value	Residential Area		
	Urban (%)	Rural (%)		aOR^a^	95% CI	p-value
**Rotterdam Criteria**						
Did not meet any Rotterdam criteria	66.7	33.6	0.000***	Ref.		
Meet only two Rotterdam criteria	16.2	20.4		2.497	(1.176,5.299)	0.017*
Meet three Rotterdam criteria	17.2	46.0		5.313	(2.698,10.461)	0.000***

Table 5 illustrates group differences in depression, anxiety, stress and insomnia between Urban and rural women diagnosed with PCOS. Considering depression, no significant difference was found (μ = 0.749), while anxiety was found with a significant difference (p < 0.01) between urban women (μ = 14.06) and rural women (μ = 17.29). Furthermore, insomnia was more prevalent in rural women (μ = 15.01), with a significant difference (p < 0.05) which indicates that anxiety and insomnia are significantly higher in rural women with PCOS.

**Table 5:** Group difference in Depression, Anxiety, Stress and Insomnia between Urban and rural Women with PCOS.

Emotional distress domain	Urban	Rural	Mann-Whitney U-test (Z)	p-value
*Depression*				
Mean (SD)	14.59 (5.759)	14.85 (5.987)	5452.000 (-.320)	0.749
Median (IQR)	16.0 (10)	14.00 (10)		
*Anxiety*				
Mean (SD)	14.06 (7.513)	17.29 (7.010)	4332.500 (-2.870)	0.004**
Median (IQR)	8.00 (14)	20.00 (14)		
*Stress*				
Mean (SD)	17.13 (8.311)	19.03 (8.435)	4830.000 (-1.720)	0.085
Median (IQR)	14.00 (14)	22.00 (14)		
*Insomnia*				
Mean (SD)	13.78 (4.741)	15.01 (5.734)	4714.000 (-1.979)	0.048*
Median (IQR)	14.00 (6)	16.00 (8)		

## 4. Discussion

Polycystic ovary syndrome (PCOS) is an extensive and under diagnosed endocrine disorder among women, which have significant impact on their physical and mental health during reproductive ages [30]. In Bangladesh, inadequate healthcare access, low awareness, and cultural burdens surrounding fertility exaggerate the psychological burden of such disorder. According to the study, about half of the participants was affected by PCOS. This result also aligns with a number of South Asian hospital-based studies that used the Rotterdam criteria which reported a high prevalence of PCOS particularly in Bangladesh and India [31,32]. Moreover, societal stigma, gender norms, and rural-urban differences further deteriorate emotional distress among affected women [33].

The findings of this study showed that women diagnosed with PCOS were around 10 times more likely to experience emotional distress compared to women without the disorder. This estimation surpasses previous studies that reported an 8.32-fold intensification in the probability of stress among PCOS patients [34]. Moreover, women experiencing stress had a 4.2-times higher likelihood of developing depression, underpinning the well-established comorbidity between stress and depression in such women. Thus, these findings are consistent with the stress theory, which posits that chronic physical conditions like PCOS works as persistent stressors that intensify psychological vulnerability, predominantly to anxiety and mood disorders [35].

In addition, sleep disturbance is another prominent mental concern that associated with diagnosed PCOS. Findings showed that more than half of women with PCOS experienced insomnia that is a much higher rate than previously reported among Polish and UK women with PCOS [22,36]. This heightened prevalence reinforced the stress process model that suggests, chronic stressors including hormonal disparities, social stigma, and physical distress may generate secondary effects like sleep disturbances. Furthermore, academic and occupational stressors, poor sleep hygiene, and a lack of mental health literacy contribute to this outcome, particularly among working women [26].

Apart from these psychosocial factors, biological mechanisms could potentially account for the elevated incidence of psychological issues in PCOS-afflicted women. PCOS is characterized by endocrine abnormalities such as insulin resistance and hyperandrogenism, which are linked to neuroendocrine dysregulation and altered neurotransmitter signaling, including decreased serotonin and dopamine activity. These factors increase susceptibility to anxiety and depression [11,34]. PCOS is often associated with persistent low-grade inflammation and dysregulation of the HPA axis, which further weakens stress-response systems and lead to mood disorders [35]. Furthermore, these hormonal abnormalities are made worse by obesity and metabolic dysfunction, which are prevalent in PCOS and have been closely associated with insomnia and other sleep disorders [12].

Beyond these biological pathways, the prevalence of PCOS is also strongly shaped by psychological responses and socio-cultural as suggested by the bio psychosocial framework [37]. In rural Bangladesh, where femininity is thoroughly linked to reproductive capability, irregular menstrual cycles and infertility frequently lead to increased stigmatization, social isolation, and internalized disgrace [38]. This study found the elevated levels of stress and anxiety observed among rural women with PCOS that internalize such expectations. Previous studies also showed the intense social pressure about body image and fertility, that make hinder their mental well-being [16]. These dynamics reflect the broader influence of symbolic interactionism that highlights how social interactions and cultural narratives shape individuals’ self-concepts, particularly when they depart from traditional social norms [39]. Considering the Rotterdam criteria, women who met two or three diagnostic criteria showed significantly higher prevalence rates for all four mental health disorders compared to those who met fewer or none of them. A previous study has shown similar findings, indicating that women diagnosed with PCOS according to the Rotterdam criteria demonstrate significantly higher levels of psychological distress [40,41].

In addition, this study highlights the crucial influence of residential location on mental health outcomes among women with PCOS, with those in rural areas experiencing more severe symptoms as well as higher levels of depression, anxiety, and insomnia compared to their urban counterparts. This is consistent with previous studies [42]. The structural strain theory asserts that such differences are result of the social structures and existing inequalities in the society due to lack of access to healthcare, education, and employment [37]. Moreover, rural health care systems typically lack the specific logistics required for dealing complex syndromes like PCOS. The unequal circulation of medical substructure further leads to late interventions, intensifying both physical symptoms and psychological strain [43]. Consequently, women with PCOS in rural Bangladesh frequently face noteworthy psychological issues as a result of inadequate reproductive healthcare, delayed diagnosis, and insufficient mental health services, all of which exacerbate the condition’s mental health burden [44]. Afterwards, the present study also found that women in rural areas exhibited a higher propensity to fulfill multiple Rotterdam criteria, indicating a more pronounced clinical manifestation of PCOS. The findings correspond with previous studies, demonstrating that women with PCOS living in rural regions displayed more severe phenotypes than those in urban areas [45].

On the other hand, concern regarding body image is found as a critical psychological stress across all BMI categories [46]. This study showed that many women are dissatisfied with their appearance, specifically due to PCOS-related symptoms such as weight gain, acne, and hirsutism. These concerns were strongly related to depression and anxiety, consistent with previous findings [47]. The social pressure and body image framework also denotes that cultural standards of fineness and femaleness marginalize women whose appearance deviates from these norms [48].

Yet, relational dynamics has critical influence on the psychological well-being of women [49]. The study showed that women in romantic relationships were 2.767 times more probable to encounter depression than those who are single. It is also revealed in previous study that married women in Bangladesh scored above the cut-off point for depression [50]. Besides, the feminist sociological theory advocates that in patriarchal societies like Bangladesh, romantic and marital relationships often augment reproductive prospects, thereby endangering women to continual inspection over their fertility [51]. Furthermore, the labeling theory suggests that such women who are deviate from cultural norms surrounding fertility may be labeled as ‘defective’ or ‘incomplete,’ resulting in assumed social stigma as products of systemic gender inequality, which ultimately deteriorating mental health outcomes [52].

### 4.1. Strength and limitations

This study offers noteworthy implications into the mental health challenges faced by women with PCOS in Bangladesh, particularly emphasizing the differences between rural and urban areas. The study utilizes validated psychometric tools like DASS-21, ISI-7, and multivariate analysis to enhance its reliability. The application of the Rotterdam diagnostic criteria adds clinical relevance. Notably, this study is among the first in Bangladesh to compare emotional distress and insomnia in PCOS patients across different geographical locations.

However, this study is not beyond limitations. There is a constraint of causal interpretations between PCOS and psychological outcomes since it is a cross-sectional design. Additionally, selection bias may have occurred since convenience sampling was used to pick individuals from gynecology outpatient departments. Compared to the general population, women who visit gynecology clinics may be more likely to experience reproductive health issues, which could result in higher prevalence estimates of PCOS and related psychological distress. However, the internal validity of comparisons across groups is unlikely to be significantly compromised because both PCOS and non-PCOS women were included. The prevalence of PCOS and psychological distress may have been higher than would have been predicted from a community-based population sample because of this recruitment strategy. In order to improve external validity, random sampling in community settings should be used in future research. This would enable more representative prevalence estimates and assist verify whether the high rates seen in this hospital-based sample are reflective of the general population. Moreover, self-reported measures may present response bias, predominantly in conservative surroundings like rural Bangladesh, where talking about mental health and reproductive issues still perceived as taboo. Yet, further research can utilize longitudinal and triangulation approaches to discover the chronological associations between PCOS and mental health. Furthermore, investigating intersectional factors including gender norms, healthcare access, and relational dynamics, across varied populations will provide deeper perceptions into socially delicate interventions for PCOS-related mental distress.

## 5. Conclusion

Polycystic ovary syndrome is a clinically significant hormonal condition affecting women of reproductive age. The rising prevalence of mental health issues is becoming increasingly concerning for Bangladesh and globally. The findings indicated a greater prevalence of psychological factors in women diagnosed with PCOS, especially those living in rural areas. Besides, a significant association was identified between PCOS and elevated levels of all four mental health factors, namely depression, anxiety, stress, and insomnia, in reproductive-aged women in Bangladesh. Furthermore, women diagnosed with PCOS who satisfied two or more of the Rotterdam criteria demonstrated notably higher levels of psychological morbidities.

In these circumstances, policymakers should implement interventions that include regular screening of psychological and physical wellbeing as integral components of PCOS treatment, particularly for rural women and those exhibiting the PCOS phenotype. Further, health ministry could plan some workshops on mental health to raise awareness and organize counseling sessions in rural parts in order to save them from such mental health problems and to use their capacity to its maximum potential. Future research should include intervention and longitudinal studies to enhance understanding of the causal pathways associated with PCOS and to develop interventions focused on improving the quality of life for women affected by this condition.

### Highlights

- Women with PCOS showed a higher prevalence of emotional distress.
- Women meeting two or three Rotterdam criteria had higher prevalence of distress.
- Rural women had higher risk of having anxiety and insomnia.
- Women who engaged in a relationship had higher chance of having depression.
- Overweight women had higher risk of experiencing depression.

## Supporting information

S1 Data Anonymized dataset used for the analysis. https://doi.org/10.5281/zenodo.17955683. (XLSX)
